# Involvement of the ocular system in hypohidrotic ectodermal dysplasia

**DOI:** 10.1186/1746-160X-8-S1-I3

**Published:** 2012-05-25

**Authors:** Thomas Kaercher

**Affiliations:** 1 Independent consultant, Heidelberg, Germany

## 

X-linked hypohidrotic ectodermal dyplasia (XLHED) is the most common form of ectodermal dysplasia. It often presents with ocular symptoms, already in early childhood. Multiple ophthalmological tests are available, but in early childhood only tests of lower invasiveness can be applied. We have evaluated tear film tests and ocular surface staining as screening methods in pediatric patients with signs of ectodermal dysplasia. These tests may also be used in adults with confirmed XLHED to determine the severity of ocular surface disease.

Twelve children and 14 adults with XLHED were subjected to a panel of tests including the ocular surface disease index (OSDI), non-invasive measurement of tear film break-up time (NIBUT), osmolarity, Schirmer test, lissamine green staining, fluorescein staining, meibography and infrared thermography. Sensitivity and specificity were determined for single tests and selected test combinations. For adults with XLHED, OSDI, NIBUT and osmolarity were the best single routine tests (sensitivity between 84.6% and 85.7%; specificity of 100%). Their combination increased the sensitivity to 92.8%. Meibography yielded optimal results (100% sensitivity and specificity). Infrared thermography revealed a typical pattern for XLHED. In children with XLHED, NIBUT or OSDI were the most convincing single tests (sensitivity of 90.9% and 83.3%, respectively; specificity of 100% each), combination of which increased the sensitivity to 100%. More invasive tests such as meibography and infrared thermography led to good results if they were tolerated.

Tear film tests can help to establish an early diagnosis in individuals with suspected XLHED, even before genetic test results are available. Meibomian gland disorder and resulting hyperevaporative dry eye are typical features of XLHED. Once the diagnosis is made, tear film tests are an important instrument to establish an effective therapy of dry eye disease.

